# Impact of the deep squat on articular knee joint structures, friend or enemy? A scoping review

**DOI:** 10.3389/fspor.2024.1477796

**Published:** 2024-11-19

**Authors:** Andrés Rojas-Jaramillo, Daniel A. Cuervo-Arango, Juan D. Quintero, Juan D. Ascuntar-Viteri, Natalia Acosta-Arroyave, Juan Ribas-Serna, Juan José González-Badillo, David Rodríguez-Rosell

**Affiliations:** ^1^ Educational and Pedagogical Studies and Research Group (GEIEP), Corporación Universitaria Minuto de Dios, Medellín, Colombia; ^2^ Universidad Corporación en Estudios de la Salud (CES), Area of Epidemiology, Medellín, Colombia; ^3^ Methodology and Research Department, Indeportes Antioquia, Medellín, Colombia; ^4^ Research Division, Dynamical Business & Science Society—DBSS International SAS, Bogotá, Colombia; ^5^ School of Health Sciences, Universidad Pontificia Bolivariana, Medellín, Colombia; ^6^ Department of Medical Physiology and Biophysics, University of Seville, Seville, Spain; ^7^ Department of Sport and Computer Science, Universidad Pablo de Olavide, Seville, Spain; ^8^ Physical Performance & Sports Research Center, Universidad Pablo de Olavide, Seville, Spain; ^9^ Research, Development and Innovation (R&D+I) Area, Investigation in Medicine and Sport Department, Sevilla Football Club, Seville, Spain

**Keywords:** injury, physical performance, osteoarticular health, range of movement, resistance training, sport-related actions, muscle strength

## Abstract

**Background:**

The squat exercise has been shown to improve athletic performance. However, the use of the deep squat has been questioned due to claims that it may cause knee joint injuries. Therefore, the purpose of this scoping review was to synthesize existing literature concerning the impact of deep squats on knee osteoarticular health in resistance-trained individuals.

**Methods:**

This study adhered to the Preferred Reporting Items for Systematic Reviews and Meta-Analyses for Scoping Reviews (PRISMA-ScR) guidelines. The original protocol was prospectively registered in Figshare (https://doi.org/10.6084/m9.figshare.24945033.v1). A systematic and exhaustive search was conducted in different databases: PubMed, Scopus, Web of Science, and SPORTDiscus. Additional searches were performed in Google Scholar and PEDro. The main inclusion criteria were the following: (1) Articles of experimental, observational, or theoretical nature, including randomized controlled trials, longitudinal studies, case reports, integrative reviews, systematic reviews, and meta-analyses(Primary studies were required to have a minimum follow-up duration of 6 weeks, whereas secondary studies were expected to adhere to PRISMA or COCHRANE guidelines or be registered with PROSPERO; (2) Peer-reviewed articles published between 2000 and 2024; (3) Publications written in English, Spanish and Portuguese; (4) Studies reporting the effects of deep half, parallel or quarter squats on the knee or evaluating squats as a predictor of injury.

**Results:**

The keyword search resulted in 2,274 studies, out of which 15 met all inclusion criteria. These 15 studies comprised 5 cohort studies, 3 randomized controlled trials, 4 literature or narrative reviews, 1 case study, and 2 systematic reviews, one including a meta-analysis. Overall, the risk of bias (ROB) across these studies was generally low. It is worth noting that only one study, a case study, associated deep squats with an increased risk of injury, the remaining 14 studies showed no negative impact of deep squats on knee joint health.

**Conclusion:**

The deep squat appears to be a safe exercise for knee joint health and could be included in resistance training programs without risk, provided that proper technique is maintained.

## 1 Introduction

Resistance training (RT) is considered as a fundamental and crucial component for enhancing strength, speed, muscular endurance, movement velocity and hypertrophy (1–6). Consequently, it should hold a pivotal role in all training regimens, contributing significantly to the increase the physical performance in various sports disciplines, and also to the improvement of physical condition, mental health and general well-being in various populations (1, 2, 7–11). Regardless of the person undergoing training, and especially in the sports field, the primary aim of RT should be to achieve a greater application of force within progressively shorter time frames (1, 12), which means an improvement in the rate of force development and implies an increase in movement velocity against a given absolute load (12). Among the widely used exercises aimed at lower limb strengthening and hypertrophy across populations is the back squat, either alone or in combination with other exercises (5, 13–20). This is due to the important association observed between the maximum strength (i.e., 1RM) of the lower-limb measured through this exercise and performance in different sports actions such as vertical and horizontal jump, linear sprint, change of direction, repeated sprint ability, tackling proficiency (21–28), the injury incidence (29), and competition-induced muscle damage (30). Notably, exclusive utilization of full or deep squat exercises during RT yields favorable outcomes in enhancing jumping, sprinting, the ability to repeat sprints, and kicking ball speed (15, 17, 31–36), which means that this exercise produces a high degree of transfer to sporting actions (37, 38).

The squat is a closed, multi-joint kinetic chain exercise involving multiple joints and significant muscle mass spanning from the erector spinae to the hip and leg extensors (18, 39). A wide variety of execution techniques have been analyzed depending on foot position (i.e., feet orientation), stance width (narrow, medium or wide), bar placement location (front- or back- squat and low- or high-bar), type of bar used (normal or safety squat bar) or footwear type (running vs. specialized weightlifting shoes) with the aim of reducing the risk of injury and increases the beneficial effects on muscle strength and development of specific sport actions (40–45). An essential consideration when exploring various squat techniques is the pivotal role of squat depth during the execution (18, 40). It is crucial to note that the depth of the squat exercise is determined by the angles formed at the knees and hips during the movement, rather than only focusing on the vertical displacement of the bar (18, 46). With a knee angulation of 180° representing full extension, squats are typically categorized based on the inner knee angle, as follows (18, 47–50): (a) *Quarter Squat:* executed within an angle range of 110°–140°; (b) *Half Squat:* performed with knee flexion between 80° and 100°; (c) *Parallel Squat:* the anterior aspect of the thigh aligns parallel to the floor, with an approximate knee angle of 60°–70°; and (d) *Deep or full Squat:* carried out until the back of the thigh and calf make contact, corresponding to knee flexion between 40° and 45°.

Exploring the acute effects of executing the squat exercise at varying degrees of knee flexion, it has been noted that the deep squat yields greater maximum moments of force in the knee extensors, heightened levels of muscle activation, relative muscle effort, and maximum execution velocity for a given relative load compared to the parallel or half squat (31, 39, 51–53). In terms of medium- to long-term effects, previous research has indicated that performing the deep squat may lead to superior strength gains and enhancement of sports performance activities such as vertical jumps and sprints than the squat with other knee flexion ranges (31, 49, 54). However, despite these findings, controversy persists regarding the innocuousness and safety of incorporating the deep squat into RT programs. Some studies caution against the use of the deep squats, citing potential risks to osteoarticular structures, particularly the knee. Specifically, it has been suggested that the stresses induced by weight-bearing squats may be associated with patellofemoral pain syndrome, articular cartilage degeneration, and chronic knee pain (55–57). Another source of debate about squat safety is in relation to the sticking point (i.e., the point in the range of joint movement at which a greater biomechanical disadvantage occurs in order to apply force against the resistance to be overcome). In the deep squat exercise, the sticking point is identified with a critical moment during the ascent phase and occurs at approximately between 60° and 90° of knee flexion (58). Due to the increase in the moment arm at this point, the force required to continue the movement is greater (59). This factor could affect technique and lead to compensatory movements and loss of correct back posture, increasing the perception of a higher risk of injury (60). These challenges could cause failures in the stability and efficiency of the movement, increasing the load on undesired structures (58, 61).

In the 1960s, Dr. Karl Klein conducted a sets of investigations examining the effects of the deep squat on knee ligaments (62, 63). Initial studies, involving cadavers and later with athletes across various disciplines, indicated that activities such as deep knee bends, duck walk, jumping squats, or laterally rotating of the knee, could result in excessive elongation of the medial collateral and lateral collateral ligaments of the knee, potentially increasing injury risk (62). Subsequently, this same author (63), after analyzing the practices of weightlifters and measuring knee joint ranges of motion compared to subjects who did not perform resistance training or exercises with high knee flexions, concluded that deep squat performance led to anterior cruciate ligament instability and general knee instability, advising against its use to prevent ligamentous weakening and recommending limiting squats to no more than half depth (63). Additionally, other publications suggested that excessive knee flexion caused medio-lateral and anteroposterior instability due to heightened shear forces on the knee joint, potentially compromising speed and agility (64–66). A more recent review (59) suggested that deep squats may impose higher compressive forces on the knee, raising concerns about joint health. However, the conclusions of these studies (59, 62, 63) should be interpreted with caution because the evidence is still limited and there is no conclusive data that allows establishing a direct relationship (i.e., cause-effect relationship) between deep squat performance and increased injury rates.

Contrary to previous studies, other researchers (14, 67) showed that incorporating deep squats into training regimens does not seem to affect knee stability. In more recent research (31, 54, 68) comparing the effect of squat exercise with varying knee flexion degrees during training periods of 6–10 weeks, in addition to showing greater improvement in strength gains and muscle hypertrophy, no increased risk of injury was observed when participants performing the deep squat exercise. However, and despite the lack of clear scientific evidence on this association between greater squat depth and increased risk or incidence of injury, it is common to hear in training jargon that the deep squat is harmful to the knee joint.

Various epidemiological studies examining injury rates have consistently identified the knee joint as the most commonly affected area across the general population, including professional athletes, amateur athletes and sedentary individuals (69–71). Among the commonly injured structures is the anterior cruciate ligament (ACL) rupture, which often results in significant functional instability (69–71). In the United States alone, approximately 250,000 ACL injuries occur annually, with 125,000–175,000 cases, requiring surgical reconstruction. Despite considerable advancements in reconstruction techniques and evolving rehabilitation strategies, several aspects surrounding ACL injury management remain under debate (72, 73), including the timing of introduction, the appropriate start time, and the recommended depth for the squat exercise (74, 75). Consequently, concerns have arisen among coaches, athletes, and other users regarding the safety of deep squats, fueled by the high incidence of knee injuries. However, as mentioned above, existing evidence does not definitively link this type of exercise to adverse effects on knee health (76). Moreover, studies supporting these concerns primarily focus on compressive forces, without considering injury incidence or prevalence (59). On the other hand, recent research has underscored RT as the most effective approach for preventing injuries to musculoskeletal structures, including muscles and tendons (77), and in several of those studies the deep squat is used during the training process (3, 5, 17, 19, 31, 33, 36, 49, 78, 79).

Therefore, aiming to address this inquiry comprehensively, the main objective of this scoping review was to synthesize existing literature and identify gaps or potential limitations in knowledge concerning the impact of deep squats on knee osteoarticular health in resistance-trained individuals. Additionally, we have included a Supplementary Material that provides data on: (a) contact surfaces between femoral condyles and the tibial plateau derived from cadaver dissections, and (b) estimates of the applied and supported forces under specific weights, employing a free body diagrams. These analyses aim to furnish objective data facilitating a deeper comprehension of how knee flexion during squat exercises may influence knee osteoarticular health.

## 2 Methods

This study adhered to the Preferred Reporting Items for Scoping Reviews (PRISMA-ScR) guidelines established by the EQUATOR Network in 2018, serving as the pre-established protocol for conducting scoping reviews (80). The methodology was further refined by integrating the latest recommendations from the Joanna Briggs Institute (JBI) for scoping reviews, optimizing the process of problem formulation, literature search, evaluation, analysis, and presentation of findings to ensure systematic and scientifically robust review procedures (81–83). The study has been recorded in figshare with the DOI https://doi.org/10.6084/m9.figshare.24945033.v1.

### 2.1 Eligibility criteria

The following inclusion criteria were applied to this scoping review:

1. Articles of experimental, observational, or theoretical nature, including randomized controlled trials, longitudinal studies, case reports, integrative reviews, systematic reviews, and meta-analyses. Primary studies were required to have a minimum follow-up duration of 6 weeks. Secondary studies were expected to adhere to PRISMA or COCHRANE guidelines or be registered with PROSPERO.
2. Peer-reviewed articles published between 2000 and 2024.
3. Publications written in English, Spanish and Portuguese.
4. Studies reporting the effects of deep squats on the knee or evaluating squats as a predictor of injury.

Exclusions comprised studies not constituting original research (i.e., editorials, notes, dissertations, etc.), however, review articles were included to provide a broader perspective on the existing literature, as long as they met the established methodological criteria; studies involving populations with knee joint pathologies (i.e., chondromalacia, meniscus tears, ligament strains, cartilage damage); and/or those exhibiting methodological deficiencies such as inadequate control over squat depth.

### 2.2 Search strategy

During the search strategy, the extension of the PRISMA statement for reporting literature searches in Scoping Reviews (PRISMA-ScR) was taken into account as a reference guide (84, 85). Initially, we framed the following question within the PICO framework: “How does the practice of deep squats during RT affect the osteoarticular health of the knee in active individuals?”

To survey the existing literature, PubMed, Scopus, Web of Science, and SPORTDiscus were chosen as primary databases. Additionally, Google Scholar and PEDro were manually searched to identify supplementary articles. Utilizing the selected databases, we employed the following search algorithms to identify relevant articles for the review. PubMed; Scopus; Web of Science; SPORTdiscus: (“full squat” OR “Deep squat” OR “Squat”) AND (“knee lesions” OR “knee risk of injury” OR “injury” OR “illness” OR “knee injuries” OR “ACL injury” OR “Osteoarthritis” OR “Meniscus tear”). The filter “all fields” was applied to the search. Additionally, manual searches were conducted on Google Scholar and PEDro using keyword combinations. This search was conducted during the first week of October 2023 and then, a new search was carried out in September 2024. An additional search was conducted in grey literature databases, including doctoral theses, unpublished reports, and conference proceedings. No additional studies meeting the established inclusion criteria for this scoping review were identified. This search was carried out to ensure comprehensive coverage of the topic and was included in the manually reviewed databases.

### 2.3 Study registration

To identify ongoing trials during the scoping review development, the following clinical trial registries were searched: ClinicalTrials.gov, ISRCTN Registry (www.controlled-trials.com), Australian New Zealand Clinical Trials Registry (www.anzctr.org.au), and University Hospital Medical Information Network Clinical Trials Registry (www.umin.ac.jp/ctr), or be registered with PROSPERO.

### 2.4 Study selection and data extraction

Two authors (JDQ and JDA) independently conducted searches across designated databases for eligible articles. Publications meeting the inclusion criteria proceeded to the data analysis and synthesis stages. A table was created to report the findings and compare the key results, including citation/country, study type, design, participant characteristics, objectives, methodology, results, and conclusions. Any discrepancies were identified and resolved through discussion with the other authors, if necessary. The study selection process took place in November and December 2023, with a subsequent search in September 2024 that did not turn up new findings.

### 2.5 Risk of bias analysis

The Joanna Briggs Institute (JBI) Critical Appraisal Tool was used to assess the methodological quality and risk of bias in the included studies, depending on the study design. Below are the key elements of the evaluation by study type:

*Cohort Studies:*

• Were the two groups comparable at the start of the study?
• Were the exposures measured similarly and validly in both groups?
• Were confounding variables identified and adjusted for?
• Were the groups comparable in terms of exposure?
• Were the participants followed for a long enough time to observe outcomes?

Randomized Controlled Trials (RCT):

• Was randomization clearly described?
• Were the groups comparable after randomization?
• Was allocation concealment adequate?
• Was there appropriate blinding of participants, treatment administrators, and outcome assessors?
• Were missing data handled appropriately?

*Narrative Reviews:*

• Was the objective of the review clearly stated?
• Were the included studies appropriate for the research question?
• Were comprehensive literature searches conducted?
• Was the quality of the included studies critically appraised?
• Was an adequate synthesis of the results provided?

*Systematic Reviews:*

• Is the research question or objective of the review clearly defined?
• Were the inclusion criteria clear and appropriate for the research question?
• Was a comprehensive search conducted across multiple databases?
• Was grey literature included in the search?
• Was a valid tool used to assess the quality of the included studies?
• Was data extraction conducted appropriately and consistently?
• Was an adequate synthesis of the results performed, and were differences between studies accounted for?
• Was publication bias assessed, for example, using funnel plots or statistical analysis?
• Are the conclusions supported by the evidence presented in the review?

### 2.6 Bibliometric analysis

The current state of research and trends in the relationship between deep squats and knee osteoarticular health were analyzed using VOSviewer v1.6.19 software (Centre for Science and Technology Studies, Leiden University, Leiden, Netherlands) and Excel 2013 for bibliometric analysis and visualization. Initially, a search was conducted in the SCOPUS database using the following search strategy: (“full squat” OR “Deep squat” OR “Back squat”). Subsequently, the CSV data (comma-separated values) were entered into the VOSviewer software to generate two maps: a “keyword co-occurrence” overlay visualization and an “author co-citation” network map. The co-occurrence map reflects how many times two or more key terms appear together in the same set of publications, while the co-citation map shows how often two authors are cited together in other articles. It is worth noting that before creating the maps, repeated words were filtered out to avoid interference with the visualization and interpretation of the results. The graphs reflect the frequency with which two or more keywords or authors (co-occurrence and co-citation) appear together in a single document or set of scientific publications. This helps identify relationships and patterns among the investigated concepts, in this case, related to the risk of musculoskeletal injuries associated with deep squats. Additionally, the strength of association, which indicates the robustness of the link between two elements (whether keywords or authors) is considered, based on how often they co-occur or are co-cited in the same source. The greater the strength of association, the stronger the relationship between them. The size of the nodes in the graphs is proportional to the frequency of occurrence of terms or authors, visually highlighting their relevance in the analysis.

## 3 Results

**Figure 1:**
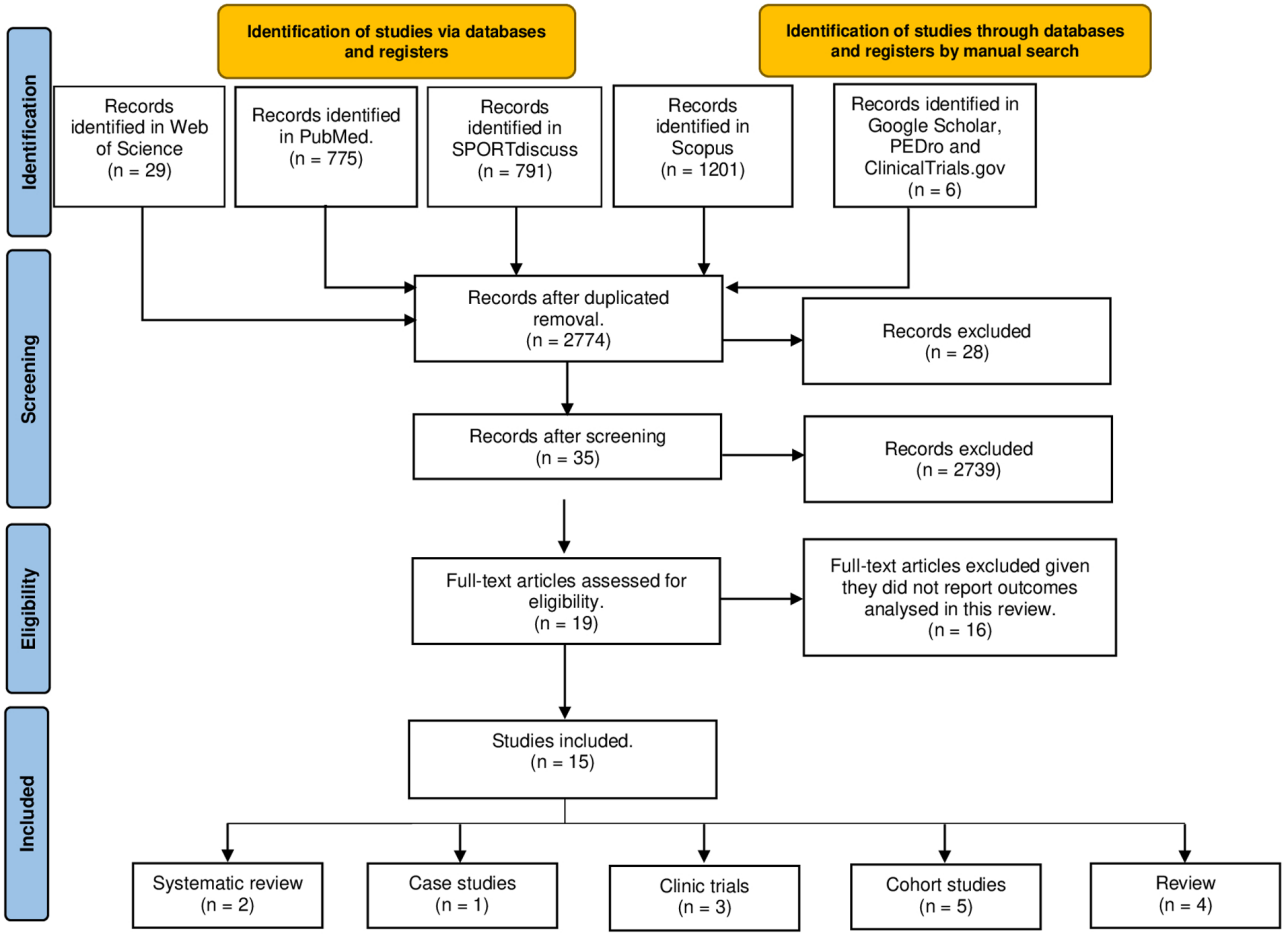
PRISMA flow diagram representing the flow of information through the different phases of the scoping review.

Following the execution of search algorithms employing Boolean operators and free language terms, a total of 2,274 references were retrieved. Subsequently, we underwent the publication selection process, which involved filtering by date, article type, and full-text availability, resulting in the identification of 35 potentially eligible studies. After evaluating abstracts, full texts, and meticulous assessment for adherence to inclusion criteria, 15 articles were excluded. A total of 15 studies met the pre-established requirements (29, 31, 48, 49, 51, 59, 68, 73, 76, 86–91). Given that this scoping review adheres to the parameters outlined in the PRISMA guidelines, Figure 1 depicts a flow chart illustrating the literature search process.

After completing a comprehensive literature review, 15 relevant studies were identified, categorized as follows: 2 systematic reviews, one including a meta-analysis, 4 literature or narrative reviews, 3 randomized controlled trials, 5 cohort studies, and 1 case study. These findings highlight the importance of conducting a detailed analysis of the impact of deep squats on knee osteoarticular health.

### 3.1 Risk of bias

**Figure 2:**
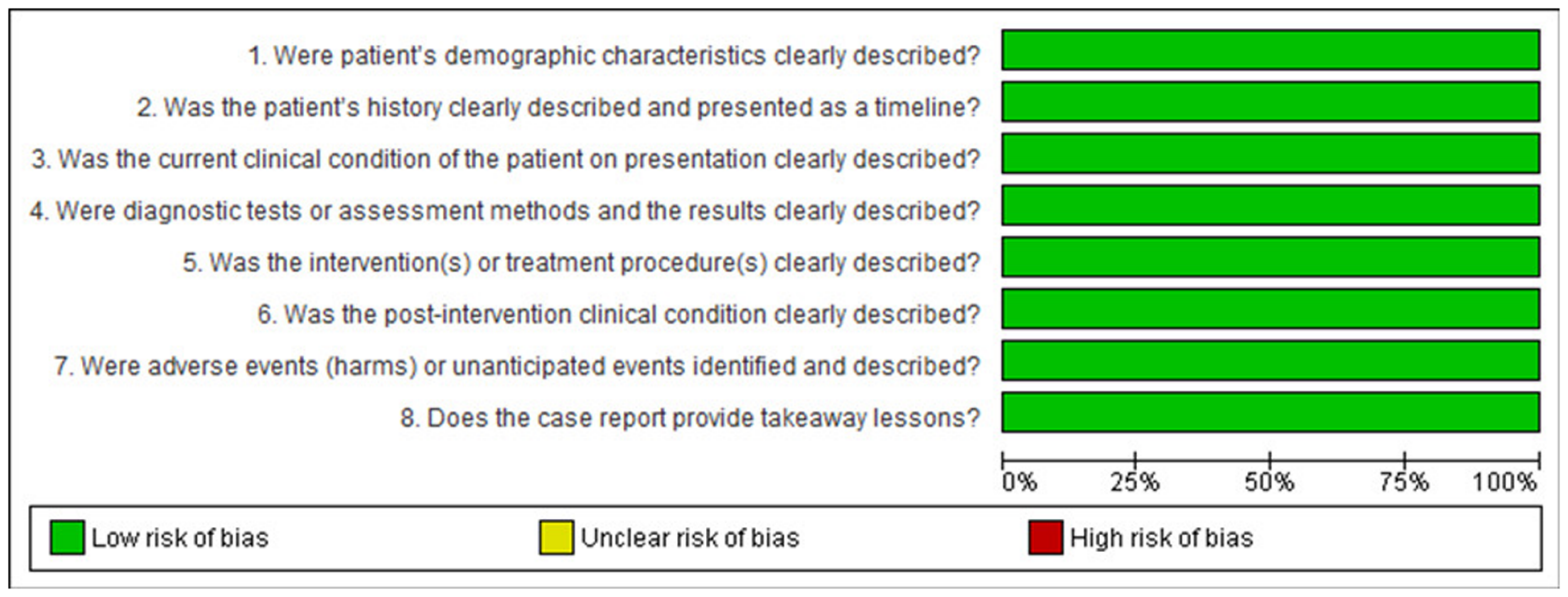
Assessment of risk of bias in the case study included in the review.

**Figure 3:**
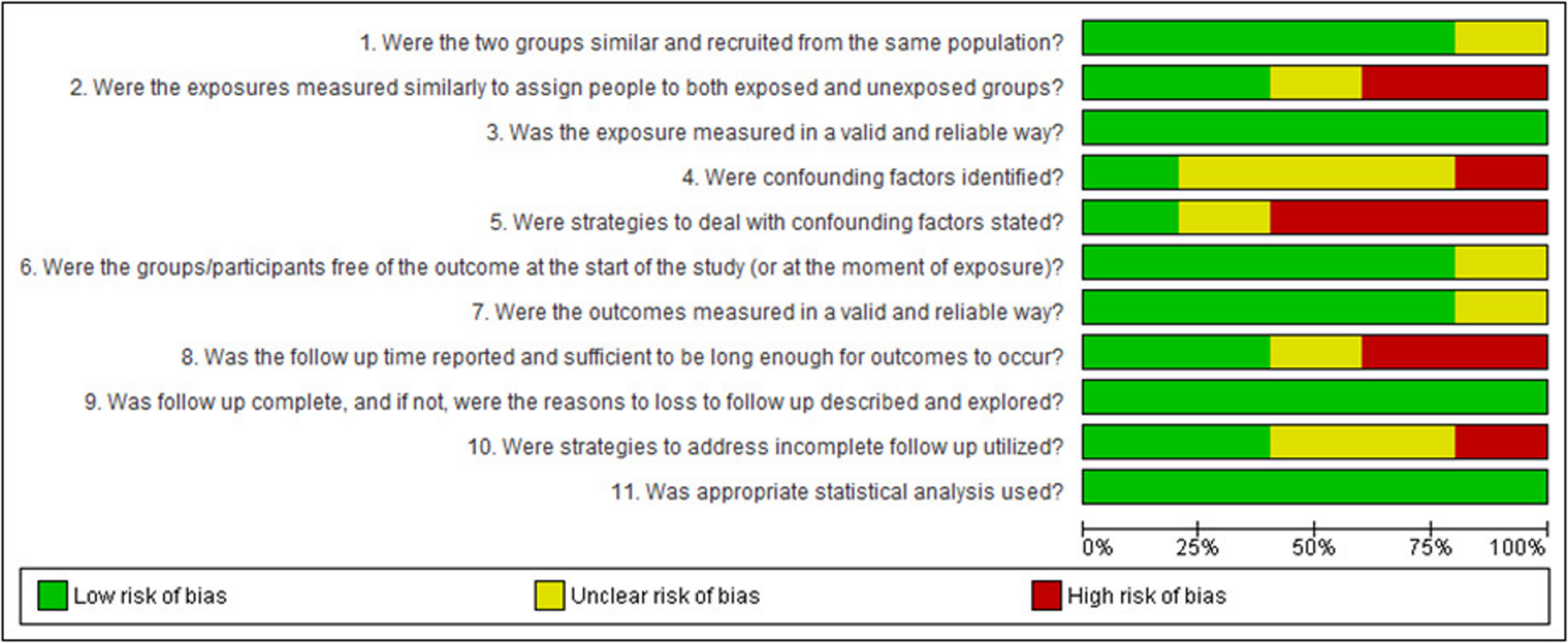
Assessment of risk of bias in the cohort studies included in the review.

**Figure 4:**
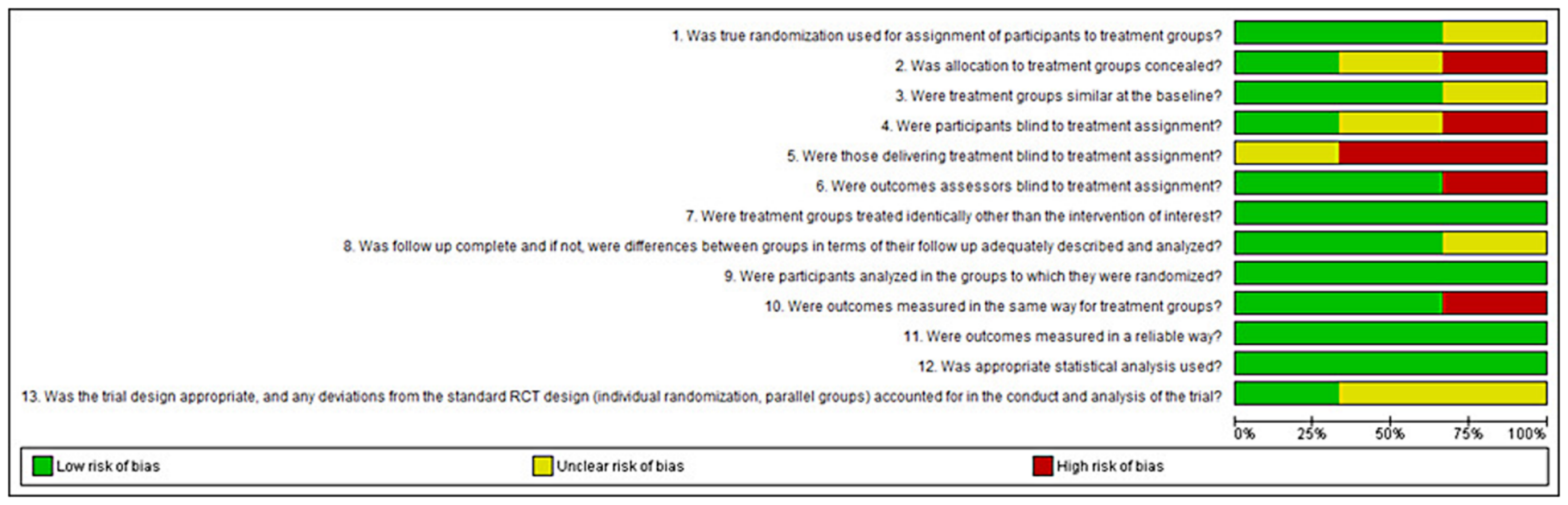
Assessment of risk of bias in the randomized controlled trials included in the review.

**Figure 5:**
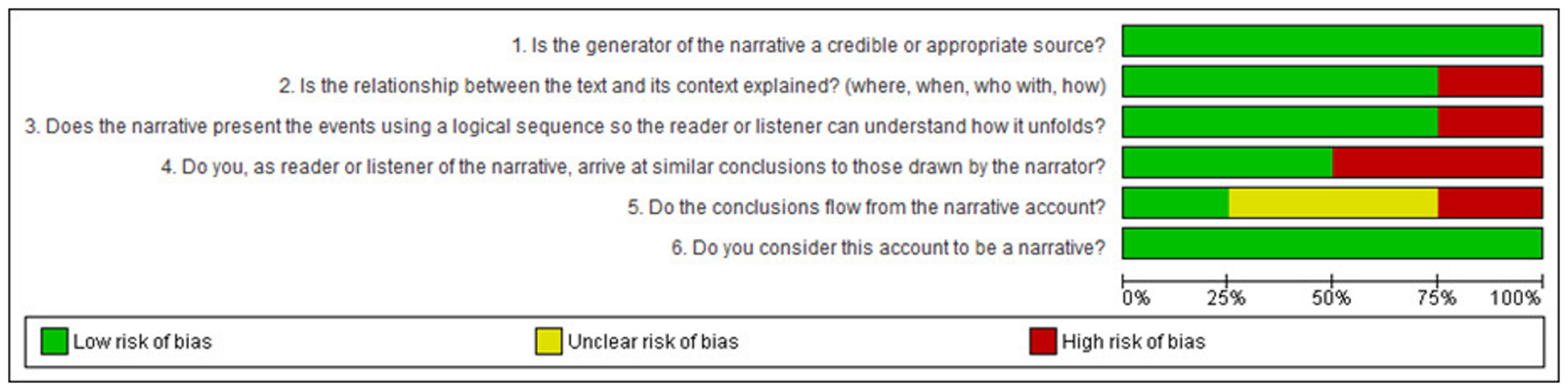
Assessment of risk of bias in the narrative reviews included in the review.

**Figure 6:**
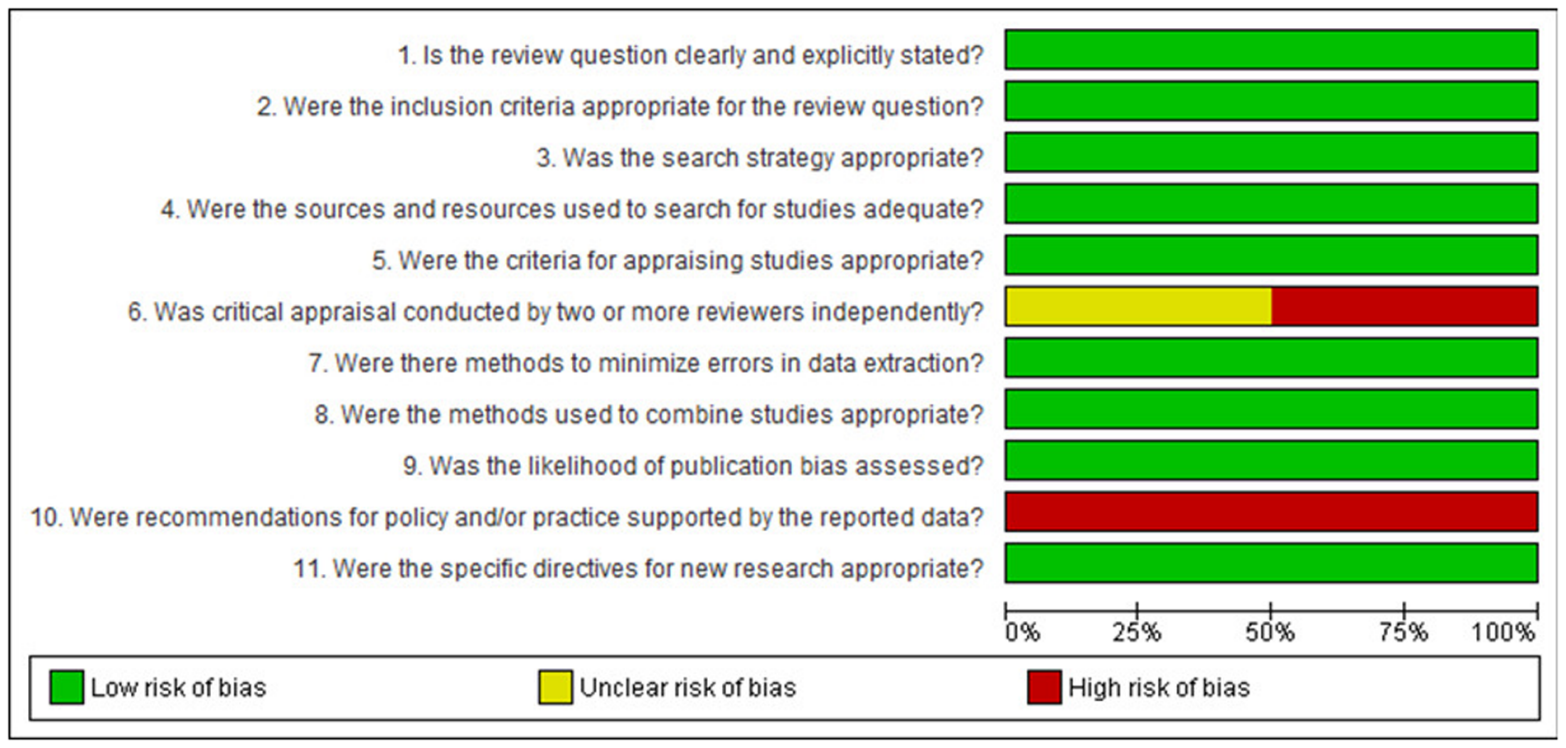
Assessment of risk of bias in the systematic reviews included in the review.

Bias assessment was conducted based on study type, with categorization into case studies (Figure 2), cohort studies (Figure 3), randomized controlled trials (Figure 4), narrative reviews (Figure 5), and systematic reviews (Figure 6), following the guidelines provided by JBI (https://jbi.global/critical-appraisal-tools). The graphs illustrating bias risks for each study are presented below.

### 3.2 Individual study findings

Table 1 provides a summary of the key characteristics (reference, study type, objectives, design, instruments and procedures, main findings, and conclusions) of the studies included in the present review.

**Table 1:** Summary of the key characteristics (reference, study type, objectives, design, instruments and procedures, main findings, and conclusions) of the studies included in the present review.

Reference	Type of study	Objectives	Study design	Instruments/procedures	Main findings/conclusions
Bloomquist et al. (68)	Randomized controlled trial	To investigate whether deep squat and half squat exercises elicited distinct effects on specific adaptations in the anterior thigh muscles and patellar tendon, as well as on jumping performance	Training period: 12 weeks *N* = 17 (M: F/17:0). Half squat group (*n* = 9) Deep squat group (*n* = 8) Sports science students Age: 23–25 years	Two types of squats were performed: deep (0°–120° of knee flexion) and half squat (0°–60° of knee flexion). Strength was assessed using 1RM, maximum isometric strength of the knee extensors was measured at various knee angles, CSA of the thigh muscles and patellar tendon was determined using MRI. Lean body mass was evaluated via DEXA scans, Muscle architecture was examined using vastus lateralis ultrasound. Collagen synthesis in the patellar tendon were assessed through pre- and post-intervention microdialysis, as well as SJ and CMJ	Heavy load FS training resulted in significant improvements in CSA, lower Lean body mass, maximum isometric strength, and SJ performance compared to HS. Both groups demonstrated improvements in 1RM and CMJ tests, indicating that resistance training in both variants is advantageous
Bryanton et al. (51)	Cohort study	To determine RME during the squat exercise. Specifically, the effects of barbell loading and squat depth on the RME of the hip extensor, knee extensor, and ankle plantar flexor were evaluated.	*N* = 10 (M/F: 0/10) Age: 22.5 ± 2.1 years College students with experience in resistance training	Squats (50%–90% 1RM) were executed within a motion analysis laboratory to assess the net joint moments of the hip extensors, knee extensors, and ankle plantar flexors. Additionally, maximum isometric strength was measured concerning the joint angle of these muscle groups. Relative muscle effectiveness was determined by calculating the ratio of net joint moments to the maximum voluntary torque at the corresponding joint angle	The impact of barbell loading on the RME of the ankle plantar flexors is more significant compared to squat depth, whereas the reverse holds true for the knee extensors. Both barbell load and squat depth influence the RME of the hip extensors
Bunn et al. (87**)**	Systematic review	To assess the correlation between full squat performance and the risk of musculoskeletal injury	*N* = 5 prospective articles regarding FS as a risk classification test for musculoskeletal injuries. Various databases were utilized from 2016 to 2018	A variety of studies were examined by gathering data from databases such as MEDLINE, SciELO, SCOPUS, SPORTDiscuss, CINAHL and VHL in December 2016. Information was extracted on participants, sample size, musculoskeletal injuries, follow-up, study design, and outcomes, followed by a risk of bias analysis	Detection of movement dysfunctions in FS exercise may act as a predictive indicator for the risk of musculoskeletal injuries in individuals participating in physical activities
Bunn et al. (86)	Cohort study	To investigate the correlation between movement patterns assessed by the Dynamic Movement Assessment and the likelihood of musculoskeletal injuries in Navy cadets	Investigation period: 1 year. *N* = 240 (M: F) N/A Navy cadets Both sexes. Age: 18–22 years	Participants completed various movement patterns in the Dynamic Movement Assessment (FS, step up and SLS). Participants were classified according to the risk of developing lesions at high, moderate, medium, or low risk. Predictive associations between lesions and risk classification were examined using logistic regression analyses	No correlation was observed between movement patterns and musculoskeletal injuries in Marine cadets. However, attending a non-military high school significantly elevated the risk of musculoskeletal injuries, ranging from 3.14 to 4.57 times. Moreover, a history of previous injuries was strongly associated with the occurrence of acute injuries
Bushman et al. (88)	Cohort study	To determine the association and predictive value of specific FMS tests with injury risk in physically active men	Investigation period: 6 months. *N* = 2,476 soldiers Age: 18–57 years	Data were gathered via surveys and medical histories covering overuse and traumatic injuries for six months post-evaluation. Sensitivity, specificity, PPV, and NPV were calculated, along with receiver operator characteristics to determine the AUC. Risks, risk ratios, odds ratios and 95% confidence intervals were calculated to assess injury risks	FMS demonstrated low sensitivity, PPV, and AUC across all seven tests for three types of injuries (2%–24% sensitivity, 16%–74% PPV, and 50%–58% AUC). Despite pain correlating with higher injury risk in five tests, the FMS still exhibited low sensitivity, PPV, and AUC. Therefore, caution is recommended when implementing the FMS test as a screening tool in military or similarly active populations, as it may lead to misallocation of prevention and treatment resources to individuals not at elevated risk of injury
Case et al. (29)	Cohort study	To investigate the effectiveness of using the relative strength level of Division I athletes in a back squat 1RM as a predictor of lower extremity injury	*N* = 46 football players. *N* = 25 volleyball and softball players. Injured and uninjured individuals. Age: 19–3 years old	Strength was assessed using a 1RM squat, and physical data were collected from the athletes, including their body mass (kg), height (cm), and age (years). This was done to establish a context for squat strength, with squat strength normalized relative to the body mass of each athlete	Relative strength in the squat is associated with the risk of lower extremity injuries in athletes, regardless of gender. Male athletes with a relative strength of less than 2.2 times their body weight (BW) and female athletes with a relative strength of less than 1.6 times their BW in sports such as soccer, volleyball, and softball are at a heightened risk of lower limb injuries
Eckard et al. (89)	Cohort study	To assess the correlation between bipedal squat and single-leg squat performance and the likelihood of injury in athletes	Investigation period: 3 years. *N* = 115 college athletes. Sex = N/A Age = N/A	The movement quality of both BS and SLS was evaluated. A single observer assessed the movement quality in real-time and documented any execution errors. Additionally, the number of days at risk for each athlete was calculated based on their participation status (“full,” “limited,” or “out”), considering four covariates: injury history, sports cut-off load, sex, and body mass index	There was no significant association found between poor mobility in the BS and SLS tests and the incidence of lower extremity injuries. Additionally, the tests demonstrated limited predictive ability for injuries
Escamilla (59)	Narrative review	To evaluate the biomechanics of the knee during the squat exercise	Not reported	The study assessed knee forces and their correlation with athletic performance, potential injuries, and rehabilitation	The squat produces shear forces regulated by the anterior and posterior cruciate ligaments across various degrees of knee flexion. Compressive forces at the knee are minimal between 0° and 50° flexion, indicating that this range is optimal for rehabilitation purposes. Muscle engagement intensifies with flexion, enabling partial squats to be executed without jeopardizing the integrity of healthy knees
Hartmann et al. (49)	Controlled trial	To compare the effects of various squat variants on the enhancement of 1RM and their transfer effects to CMJ, SJ, Maximum voluntary contraction, and rate of force development	Training period: 10 weeks Experimental group: *n* = 59 (M: F/36:23). Median age: 24.11 ± 2.88 years Front FS group (*N* = 20) FS Group (*N* = 20) Quarter squat group (*n* = 19) Control group (*N* = 16) sex: N/A Physical education students, most with little experience in resistance training	Quarter squat (120° knee extension on Smith's machine) and front and back full squats in the development of 1RM, their specific transfer effects at CMJ and SJ height, and maximal voluntary contraction and maximal rate of force development in an isometric leg press (120° knee extension) were evaluated. Participants followed a training program consisting of 5 sets of 2–10 repetitions of each squat variant, with 5 min of rest, 2 days a week, for 10 weeks	The front and back full squats demonstrate transfer effects that enhance performance from maximal dynamic force to the force-velocity capacity of the hip and knee extensors compared to the quarter squat. These findings challenge the notion of specific transfer effects at higher angles
Hartmann et al. (48)	Narrative review	To assess whether squats with less knee flexion (half/quarter squat) are more secure for the musculoskeletal system than deep squats	*N* = 164 scientific publications between March 2011 and January 2013 using PubMed. Sex = N/A Age = N/A	Data on knee forces during loaded and non-loaded squats were compared at different knee extension angles	Concerns regarding degenerative changes in the tendofemoral complex and the suggested elevated risk of chondromalacia, osteoarthritis, and osteochondritis associated with deep squats are unfounded
Illmeier et al. (90)	Narrative review	To examine the effects of various back squat variations on anterior knee displacement	Articles referenced in PubMed, ResearchGate, and Google Scholar from their start date to April 2022. *N* = N/A Sex = N/A Age = N/A	The analysis focused on publications exploring anterior knee displacement in the context of anthropometric or biomechanical studies related to various barbell squat movements for exercise, rehabilitation, or therapy. Only publications in German and English were included	To enhance training outcomes and minimize biomechanical strain on the lumbar spine and hip, it could be beneficial or even essential for numerous athletes to permit a degree of anterior knee displacement. Generally, restricting this natural mobility is likely not advisable for healthy, trained individuals
Kothurkar et al. (73, 73)	Case study	To compare the contact stress distributions in the patellofemoral and tibiofemoral joints at full extension and deep flexion. Additionally, a kinematic analysis was conducted to elucidate the kinematics of the deep squat	*N* = 1 adult without knee injury. Age = 33 years Body mass = 55 kg	MRI and computed tomographic scans were utilized for measurements and 3D modeling of the knee joint. A finite element model of the knee was used to analyze the stresses and movement of the joint at different degrees of flexion. Additionally, distances between landmarks on the femur and tibia were measured to assess joint kinematics in various positions, including full extension and deep flexion	Increased stresses on the knee joint in a deep squat position may result in damage to the patellar cartilage
Pallarés et al. (31)	Randomized controlled trial	To assess the impact of a resistance training program incorporating various squat depths on well-trained athletes using velocity-based resistance methods. Additionally, the aim is to investigate whether this training regimen results in injuries or discomfort for athletes over the long term	Training period: 10 weeks *N* = 53 (M:F/53:0) Resistance trained individuals FS Group: *N* = 13 (M:F/13:0) PS group: *N* = 13 (M:F/13:0) HS Group: *N* = 13 (M:F/13:0) Age = ∼23 years	The correlation between velocity and load during squats was assessed through a progressive load test up to 1RM. Tests were conducted for three types of squats (FS, HS, and PS) using a Smith machine. The load was progressively increased by 10 kg increments until the MPV reached ≤0.60 m/s. Load adjustments were individually to determine the precise 1RM. Kinematic parameters were recorded using a dynamic measurement system from the T-Force System. 20-m sprint, CMJ, and Wingate test while pain, stiffness, and physical function were assessed	FS and PS are optimal for enhancing strength and performance in well-trained athletes. However, HS yield limited results and may lead to discomfort, prompting reconsideration of the necessity to target specific improvements in angle and ROM during training
Schlegel et al. (76**)**	Narrative review	Assess various perspectives on executing a deep squat, analyze the optimal technical approach, and evaluate potential risks associated with its utilization	*N* = 48 articles as of February 2020. Full articles in scientific journals or relevant books. Sex = N/A Age = N/A	The authors conducted a literature review of available human studies on the research topic, covering discussions regarding the deep squat worldwide up to the year 2020	With an appropriate technique and in healthy individuals, there is no increased risk
Wolf et al. (91)	Systematic review and meta-analysis	To assess the effects of ROM on a variety of outcomes such as hypertrophy, strength, power, and body fat-related measures	*N* = 26 studies (*N* = >658) Sex = N/A Age = N/A PubMed/Medline and SportsDISCUS databases searched, included studies up to August 2022	Studies involving a resistance training intervention with at least two groups/conditions using variable ROM and measuring at least one outcome of interest (muscle size and strength, power, or body fat) were included. No restrictions were imposed on the date of publication	The primary model, involving all effects on all outcomes across 23 studies, demonstrated a trivial standardized mean difference (0.12; 95% CI –0.02, 0.26) favoring full over partial ROM

N/A, not applicable; M, male; F, female; FS, full squat; PS, parallel squat; HS, half squat; 1RM, one-repetition maximum; CSA, cross-sectional area; MRI, magnetic resonance imaging; DEXA, dual energy x-ray absorption; SJ, squat jump; CMJ, countermovement jump; MPV, mean propulsive velocity; ROM, range of motion; BP, bipedal squat; SLS, single leg squat; RME, relative muscle effort; PPV, positive predictive value; NPV, negative predictive value; AUC, area under the curve; FMS, functional movement screen; CI, confidence interval.

From the 15 studies incorporated, 87% (13 studies) assert the safety of deep squatting, and it does not result to an increased risk of injury (29, 31, 48, 49, 51, 68, 76, 86, 87, 89–92), while merely 2 studies highlight a plausible adverse correlation between deep squat practice and heightened injury risk (59, 72). One of these studies adopts a case study format (72), whereas the other assumes the form of a literature review (59).

### 3.3 Bibliometric analysis

For a better understanding of bibliometric analysis, it is necessary to take into account that the numbers in parentheses show the number of times a term or author appears in co-occurrence or co-citation, and the second indicates the strength of association with other elements of the analysis. As shown below, there is usually a positive relationship between greater co-occurrence or co-citation with greater association strength. In addition, the size of the nodes in the figures varies according to the frequency of appearance, which visualizes the relevance of each term or author throughout the analysis.

**Figure 7:**
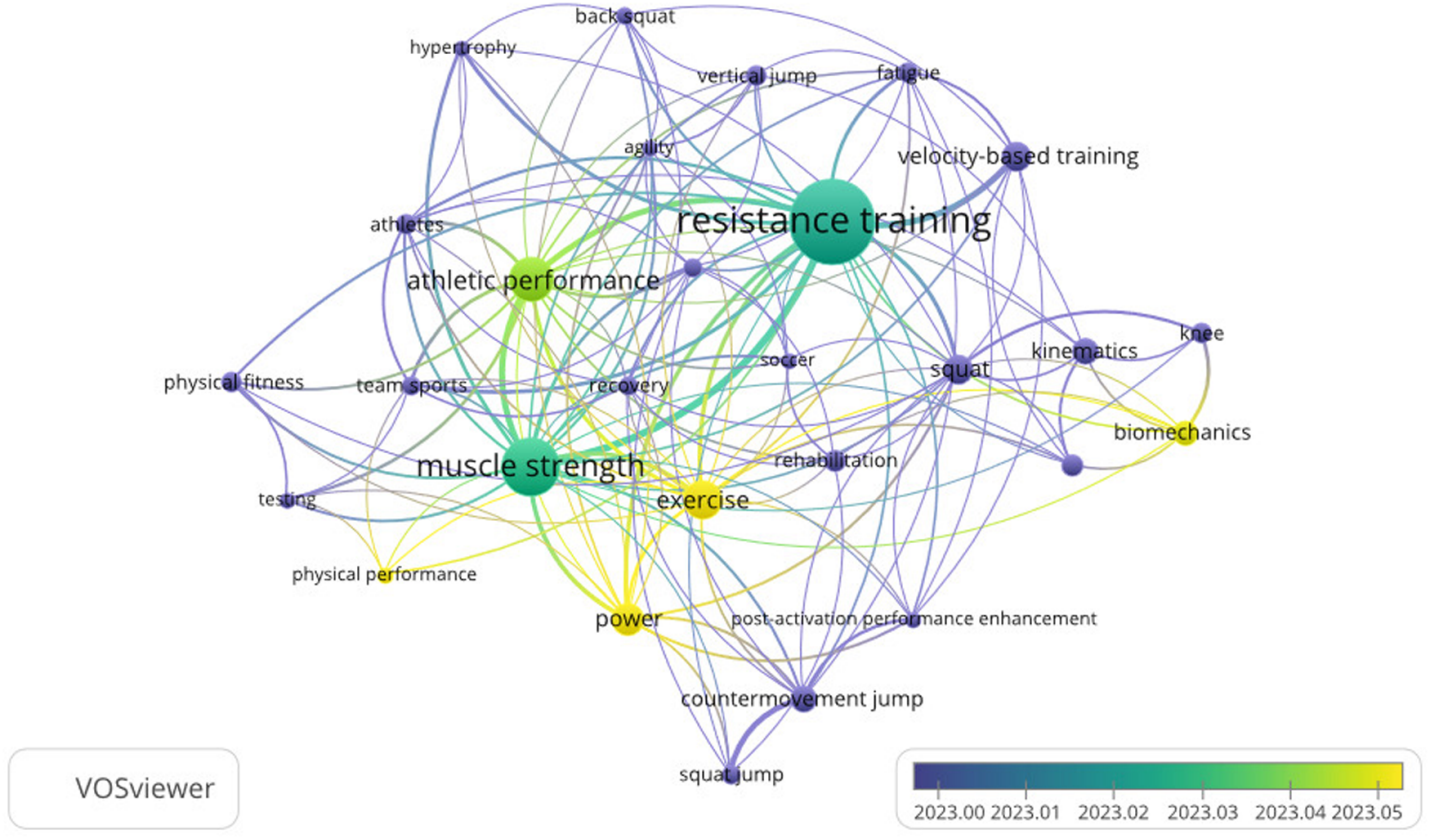
Representation of bibliometric analysis showing an overlay display of “keyword co-occurrence”. The size of the nodes represents the number of co-occurrences of the keywords in the articles and the color represents the date of publication. The connections between the nodes illustrate the degree of bibliographic reference in common among publications (https://www.vosviewer.com accessed January 06, 2024).

A trend was observed during 2023 of publications associated with the squat based on keyword co-occurrence and their strength of association: RT (127:113), muscular strength (73:95), athletic performance (49:69), exercise (39:44), and power (31:38). A close association was also observed between squatting or back squats and RT, muscular strength, athletic performance, power, velocity-based training, hypertrophy, and recovery, likely due to the scientific output associated with the application of this exercise as a physical evaluation test and/or performance enhancement (Figure 7).

**Figure 8:**
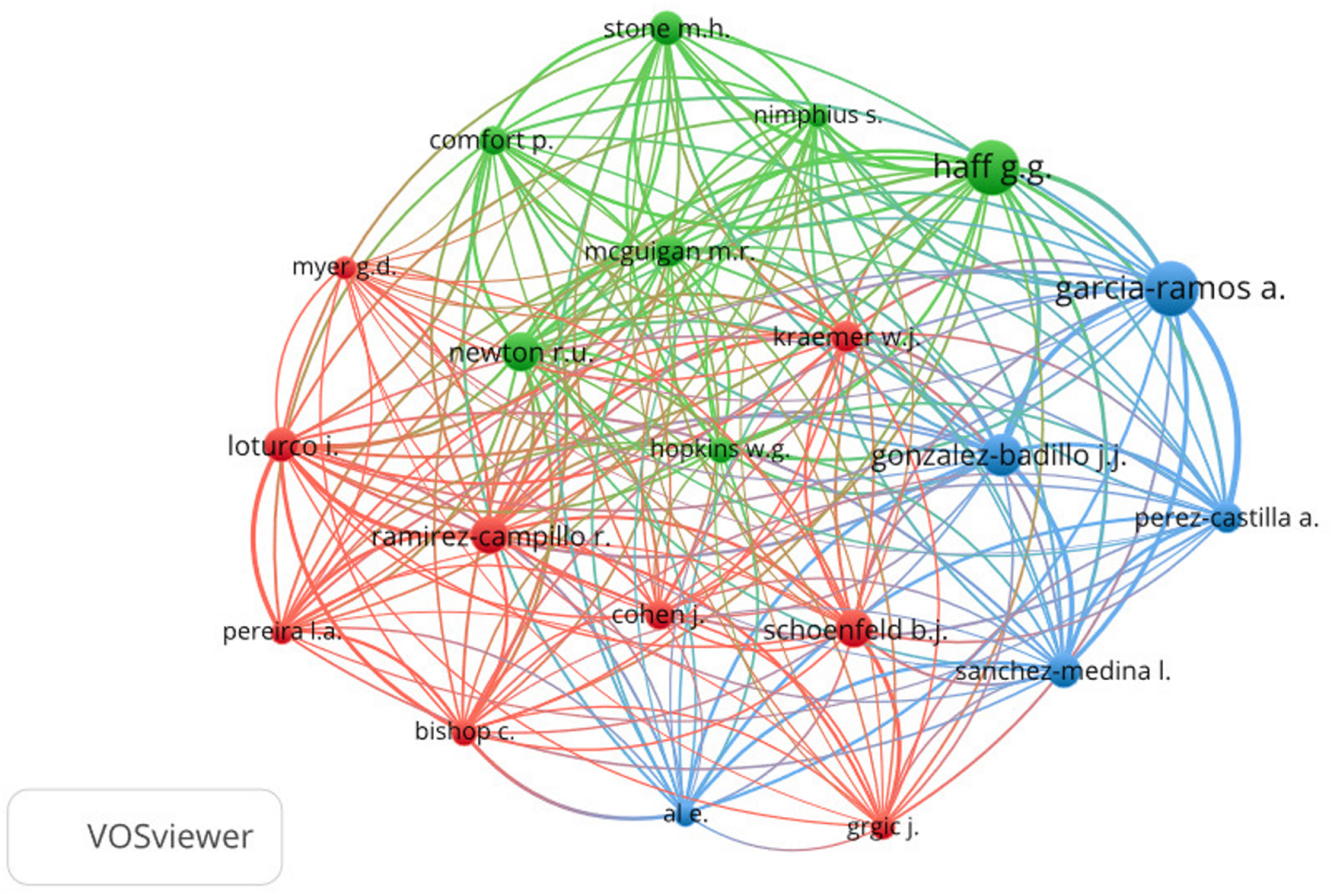
Representation of bibliometric analysis showing a network map of “co-citation of authors”. The size of the nodes represents the number of citations of the authors in the articles, and the color illustrates the degree of strength of association between authors (https://www.vosviewer.com retrieved January 06, 2024).

The co-citation analysis of authors with the most impact (at least 100 citations) in research directly or indirectly linking squatting identified the main references in various articles based on their number of citations and association strengths: Garcia-Ramos A. (269:4,144), Haff G.G. (268:3,629), Gonzalez-Badillo J.J. (198:3,152), Newton R.U. (188:2,104), Schoenfeld, B.J. (178:1,387), and Ramírez-Campillo, R. (176:2,433). These authors can be considered key theoretical sources for understanding the role of occupation from a more objective perspective across different research domains (Figure 8).

## 4 Discussion

The importance of RT using the squat exercises lies in the multiple benefits it induces on sport performance (1, 3, 5, 15–19, 31, 33, 34, 36, 91), health (2, 4), injury rehabilitation (74), body aesthetics (20), and even on cardiovascular and eye health (61, 93). However, some authors still argue that the squat exercises, particularly deep squats, may heighten the risk of knee joint injury (41, 59). To shed some light on this controversy, the objective of this study was to synthesize existing literature and identify gaps or potential limitations in knowledge concerning the impact of deep squats on knee osteoarticular health in resistance-trained individuals. The main funding was that the deep squat appears to be a safe exercise for knee joint health and could be included in RT programs for any individual without previous pathologies, provided that proper technique is maintained.

In 1984, a notable viewpoint emerged (94), attributing the belief in the injury risk associated with deep squats to a sets of studies conducted by Dr. Karl Klein in the 1960s (62, 63, 65, 66). Klein's initial research (63, 66) proposed that deep squatting leads to increased knee joint laxity, causing “micro-movements” and elevating the potential for joint surface alterations. Subsequently, after analyzing groups of athletes that included the deep squat exercises or weight-bearing jumps (powerlifter, weightlifter, or paratroopers) in their training program routines, Klein concluded that the full squat was responsible for anterior cruciate ligament and collateral ligaments laxity (63). However, Klein's conclusions were based on an unspecified “orthopedic test standard” and an unvalidated instrument for measuring knee joint laxity, neglecting the ligament stiffness or force capacity of the athletes. Consequently, the notion of deep squats causing knee osteoarticular lesions appears to stem from studies of questionable validity, due to their methods and unsubstantiated conclusions. Therefore, a more thorough examination of this critical issue was needed.

The current review highlights the absence of scientific evidence substantiating the notion that deep squatting induces knee joint damage in individuals devoid of preexisting pathology. Conversely, the limited available evidence suggests potential benefits of deep squatting in mitigating the risk of knee injuries (29, 48, 49, 68, 76). Therefore, the primary conclusion of this study was that deep squats appear to safe for osteoarticular health, and the inclusion of this exercise in training programs could allow greater physical performance gains, provided the training principles are respected, the exercise is performed correctly, and the load adjusts to the needs and requirements of the trained individuals.

The present review comprises information from 15 studies. Most of these studies evaluated the safety of the squat exercise by examining the effects of the short- and long-term interventions, specifically assessing the incidence of osteoarticular pain or injury during the exercise. Other studies have analyzed the compressive forces on the knee, hip or back, whereas some studies have conducted biomechanical analyses to identify abnormalities associated with the use of the squat exercise. As indicated, from the 15 studies incorporated, 87% (13 studies) assert the safety of deep squatting, and it does not result to an increased risk of injury (29, 31, 48, 49, 51, 68, 76, 86–91), while merely 2 studies highlight a plausible adverse correlation between deep squat practice and heightened injury risk (59, 72). Despite this negative claim about the effect of the deep squat, it is important to note that the findings of Escamilla suggest that when this exercise is executed with submaximal loads (i.e., low to moderate loads) and among individuals without underlying health concerns, the deep squat constitutes a safe exercise. On the other hand, the case study conducted by Kothurkar et al. (72, 73) proposed that increased stress on the knee joint during a deep squat could potentially harm the patellar cartilage. However, this claim is based on a hypothesis and lacks empirical evidence to support a causal relationship between increased stress and probability of injury. It is possible that greater passive stress can occur during a deep flexion compared to a parallel squat or half squat due to increased elongation of passive elastic elements (tendons, aponeurosis, and titin). However, considering: (1) enhanced likelihood of tension generation by contractile muscle elements (improved length-to-tension ratio of sarcomeres), and (2) greater moment of force for identical force exertion levels in half or parallel squats compared to full squats, it is likely that active tension and, consequently, total tension and the degree of pressure of the patella on knee structures during the half squat may equate to or even surpass those experienced during deep squats. Although this deduction seems justified, further studies are needed to support this hypothesis.

In contrast, Escamilla et al. (59) compiled several studies to conclude that the deep squat elevates shear forces, potentially increasing its injurious nature. However, subsequent research and reviews (31, 48, 49, 54, 76, 91, 95) have questioned these findings, highlighting the lack of substantial scientific evidence supporting the harmful effects of deep squats (48). This discrepancy arises from the absence of direct evaluation in these studies regarding the incidence or prevalence of injuries specifically linked to deep squat performance. Contrary to review conducted by Escamilla et al. (59), it appears that shear forces do not lead to joint injuries in the absence of ligament damage (76). In addition, it has been observed that the cartilage adapts to the load and strain imposed during deep squatting by increasing its thickness, potentially affording greater knee joint protection (48, 49). Also, in more recent studies (96, 97), it has been observed that weightlifters, despite having greater laxity joint of the knee, have stronger ligaments.

Another argument against deep squatting concerns the observation of increased compressive forces on the meniscus as knee flexion deepens, although lacking evidence suggesting harm to individuals with healthy knees (48). Moreover, Zelle et al. (98), noted that during a deep squat, contact between the back of the thigh and calf occurs, resulting in a reduction of knee compressive forces by approximately 30%. This reduction could potentially benefit knee osteoarticular health. On the other hand, several studies (51, 52) have revealed that quadriceps muscle activation is higher during deep squats compared to partial squats at the same relative load. This finding is significant as it implies that deep squats could be performed at a lower relative load compared to other ranges of knee flexion without compromising potential benefits, thus potentially reducing compressive forces on the knee (48, 51). Indeed, this argument is frequently cited as a reason to caution against the use of deep squats (48).

Additional commonly cited argument against deep squats involves the hypothesis that allowing the knees to surpass the toes during execution may lead to increased compressive forces and knee joint wear (59, 99). Nonetheless, a recent review conducted by Illmeier et al. refutes this argument, asserting that it lacks biomechanical basis. The cited review (90) concludes that it is entirely natural and acceptable for the vertical projection of the knee to extend beyond the toes during full squats. Furthermore, this movement does not appear to elevate the risk of knee joint injury (90).

As previously highlighted, the majority of studies included in this review (29, 31, 48, 49, 51, 68, 76, 86–91) support the notion that deep squats do not lead to significant osteoarticular or muscle damage, endorsing its consideration as a safe, beneficial, and recommended exercise in any training regimen. For instance, Pallarés et al. (31) reported greater knee discomfort in the group performing partial range of motion squats compared to full squats, while other studies (48, 54, 86, 87) suggest that deep squatting may help prevent knee joint injuries. Despite these positive findings, most research (18, 48, 76, 87) emphasizes that it is necessary to take into account that the “innocuousness” of this exercise on the osteoarticular structures of the hip, knee and back could be related to the ability of executing the exercise with correct and adequate technique (Table 2).

**Table 2:** Criteria for correct execution and the most common mistakes during the full-squat exercise (100).

Criteria		Description	Correct	Incorrect
Head position		Line of neck is perpendicular to the ground and gaze is aimed forward	Look ahead	Look down
Thoracic position		Chest is held upward, and shoulder blades are retracted	Chest perpendicular to the floor	Chest towards the floor
Trunk position		Trunk is parallel to tibia, while maintaining slightly lordotic lumbar spine	Trunk is parallel to tibia	Excessive anterior trunk inclination
Hip position		Line of hips is parallel to ground in frontal plane throughout squat	Line of hips is parallel to ground	Lateral hip tilts
Frontal knee position		Lateral aspect of knee does not cross medial malleolus for either leg	The knees do not move medially, in the frontal plane	knee valgus
Tibial progression angle		Knees do not excessively pass the front of the foot. Tibias are parallel to an upright torso	The knees move forward naturally	Excessive anterior translation of the knees
Foot position		Entire foot remains in contact with the ground	Entire foot remains in contact with the ground	Steepen
Movement mechanics				
Initial position	Descent	Descent	Descent	Depth
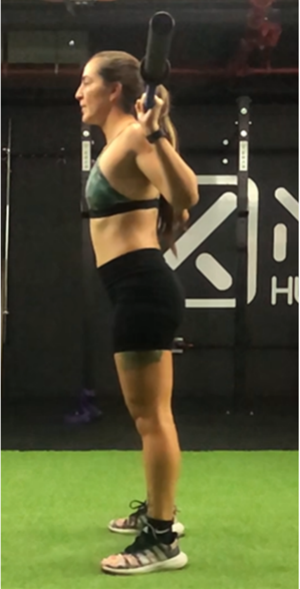	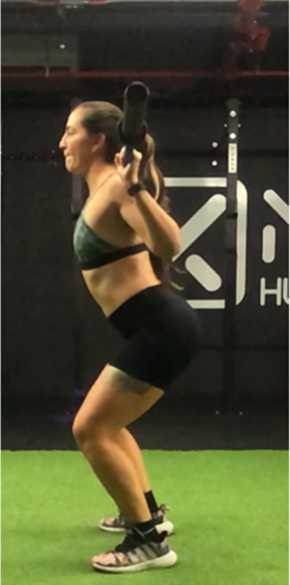	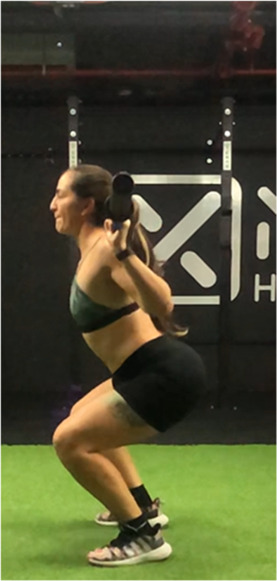	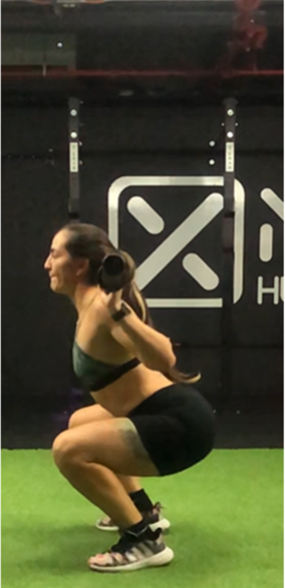	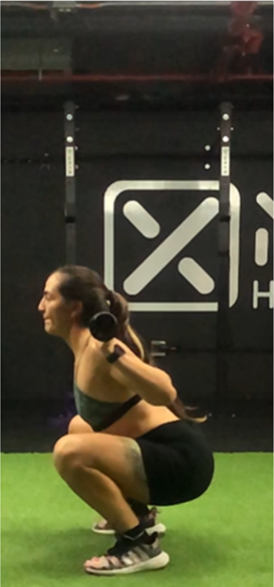

 In this sense, there are several reviews and position stand that have focused on describing biomechanical aspects related to “correct technical execution” during the deep squat exercise (18, 40, 44, 76, 100, 101).

Specifically, Myer et al. (100) conducted a comprehensive evaluation of the functional deficits and technical factors that limit performance in the back squat. In this guide, several factors affecting the proper execution of the exercise are outlined, and common errors are listed, including: (1) Head position; (2) Thoracic position; (3) Trunk position; (4) Hip position; (5) Knee position; (6) Tibial progression angle; (7) Foot position. This last point emphasizes the importance of proper ankle dorsiflexion, which is considered a limiting factor in the execution of a deep squat (102, 103). Additionally, reduced ankle dorsiflexion mobility could compromise movement mechanics, increasing the risk of knee valgus or lateral or frontal trunk tilts, raising concerns about the safety of squat execution (102, 103).

In addition to aspects related to technical execution, an often overlooked aspect, albeit crucial, is the load level, particularly the weight lifted (relative load, %1RM), and the number of repetitions performed in relation to the maximum repetitions that could be completed, as these factors may significantly impact the forces exerted on various joints (39, 51). Excessive weight could compromise technique and elevate compressive forces on intervertebral discs, hips, and knees, heightening injury risk (39, 51). Similarly, when performing sets to failure or near muscle failure could lead to accumulated fatigue that could distort the execution technique and also increase the risk of injury (3, 104). Hence, the likelihood of developing knee osteoarticular pathology appears more likely linked to the load magnitude rather than the range of motion used during full squat execution.

In the present review, several randomized controlled trials were identified (31, 49, 68) analyzing the effects of deep squats compared to squats with partial knee flexion ranges. Across all studies (31, 49, 68), greater physical performance improvements were found when conducting squats with a wider range of joint motion (i.e., deep squats), evident in both high-speed actions (i.e., vertical jump and running acceleration capacity) and muscle strength (i.e., 1RM). Furthermore, recent systematic review with meta-analyses (54, 91, 105) also concluded that strength gains were superior with deep squats compared to half, parallel or quarter squat. Notably, these studies evaluated knee pain through health questionnaires and none of them reported any knee joint damage resulting from deep squatting.

Finally, some studies incorporated in this review (88, 91, 92) use the deep squat as an indicator of injury risk. Most of these research (86–88, 92) analyze the “quality of movement” during squat execution and suggest that this aspect may not serve as a sensitive indicator or predictor for injury detection. Conversely, the relative strength ratio in deep squatting is proposed as a more effective gauge for injury risk assessment. Case et al. (29) examined the correlation between 1RM in squat exercise relative to body weight (1RM/BW, denoted as barbell squat relative strength) and the risk of lower extremity injury among athletes across various Division I sports disciplines. The findings of this study suggest that male athletes with a relative strength ratio below 2.2 and female athletes below 1.6 face an elevated risk of lower limb injuries, particularly in sports such us soccer, volleyball, and softball (29). Thus, contrary to conventional assumptions, this study underscores the importance of enhancing strength in the deep squat as a preventive measure, advocating for introducing the full squat exercise during athletes’ RT programs (29).

Although the existing literature seems to show clear evidence regarding the safety of the deep squat on the risk of injury to the knee joint, in the present review we have identified some limitations that are pertinent to be addressed. One of the main limitations is related to the duration of the interventions. Most of studies included in the present review used a relatively short duration of the interventions, typically ranging from 6 to 10 weeks. Long-term studies examining exposure to deep squats over several years would provide more comprehensive insights on this relevant issue. Secondly, many studies rely on questionnaires to assess the knee joint pain and injury risk. Therefore, it appears that future research should consider more objective and valid assessment methods to know the real influence of performing deep squats on osteoarticular health. On the other hand, although some studies report increased compression forces during deep squat as a potential risk factor for knee injury, a direct association should not be established, as there is not enough evidence to establish a cause-effect relationship between both facts. Finally, another limitation of this scoping review is the heterogeneity of the included studies, with several exhibiting moderate to high risk of bias.

The limitations presented above highlight the need to establish a research line that allows for long-term follow-ups (i.e., spanning several years) of individuals who perform the squat exercise with different ranges of motion (deep, parallel, half and quarter squat) and to analyze the possible influence on: (1) the osteoarticular health of the knee and other joints involved in the movement (hip, ankle and spine), and (2) the improvement of sports performance and healthy physical condition.

## 5 Conclusions and practical applications

To summarize, the current review concludes that deep squat is considered a safe and potentially protective exercise against injury risk, demystifying the unfounded belief that a greater knee flexion during squats could lead to a higher risk of severe osteoarticular pathology. Moreover, the beneficial effects on physical performance (i.e., strength, speed, and jumping ability) were more pronounced when training programs incorporating deep squats were performed compared to squats with other partial ranges. Based on these findings, it is reasonable to acknowledge that deep squats are a safe exercise, provided the relative loads and volumes used are not excessive for the individual's characteristics. Therefore, this exercise could and should be integrated into any RT program when there are no contraindications or limitations that hinder proper execution. In essence, the findings of the present review suggest that the problem with the squat exercise has never been the completed depth range (i.e., degree of knee and hip flexion), but rather the incorrect technique and potentially the degree or magnitude of the total load used (i.e., relative load and training volume), as could occur with any other type of exercise.

## Data Availability

The original contributions presented in the study are included in the article/Supplementary Material, further inquiries can be directed to the corresponding author.
