# Supplementary material for: Impact of the deep squat on articular knee joint structures, friend or enemy? A scoping review
Source: Front Sports Act Living. 2024 Nov 19;6:1477796. doi: 10.3389/fspor.2024.1477796 (PMC11618833; doi:10.3389/fspor.2024.1477796)
Supplement: Supplementary file 1 [file Table1.docx]

Supplementary Material

In addition to the analyses carried out in this scoping review, we undertook an exploratory investigation aiming to elucidate potential differences between the deep squat and the half squat. As part of this attempt, we constructed a hypothetical diagram depicting the condylar joint contact surface, derived from a human cadaver (Figure 1), along with approximate quantification of the forces exerted by the quadriceps femoris muscles and the load borne by the patellar tendon during both the full squat (Figure 2) and the half squat (Figure 3). Although data on force vectors in deep squats with varying loads and flexion degrees are available, information on friction surface or joint contact between the femoral condyles and the tibial plateau is lacking. To address this gap, we conducted quantitative estimations utilizing a cadaveric knee specimen sourced from the Department of Anatomy, Faculty of Medicine, Universidad de Sevilla, to approximate the femoral condylar surface (Figure 1). According to the measurements obtained shown in Figure 1, the estimated contact surface of the lateral condyle is 1365.85 mm^2^, while that of the medial condyle is 1217.45 mm^2^, and the estimated total surface is 2583.3 mm^2^.


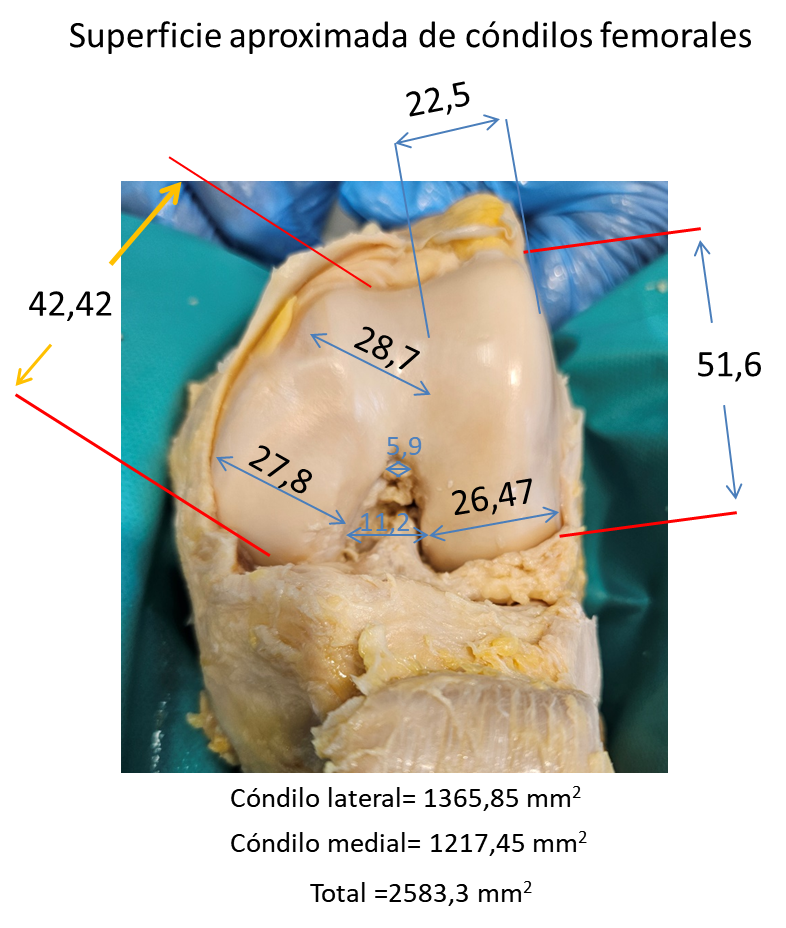


**Figure 1**. Axial view of the femoral condyles in a human knee. The estimated contact surface of the lateral condyle is 1365.85 mm^2^, while that of the medial condyle is 1217.45 mm^2^, and the estimated total surface is 2583.3 mm^2^. The measurements presented do not consider the curvilinear deformations of the structures, thus resulting in an underestimation of the total surface area. The intercondylar notch and the anterior reflection of the patellar tendon are visible in the image.

Furthermore, we developed a free body diagram incorporating a joint representation wherein a tibia is depicted alongside its corresponding foot, while a femur is positioned with its distal end situated over the knee joint, and the percentage of body weight applied to the proximal end of the femur is indicated. Although during the eccentric and concentric phase of the full-squat and half-squat movement there is a forward displacement movement of the tibia, we have simplified the model by fixing the tibia to make the system more understandable and simplify the calculations of the force vectors. The quadriceps femoris muscles are represented as a unit (Fc) with their bony insertion 0.06 m from the point (i.e., center of mass) where the percentage of body weight corresponding to a 70 kg subject's body is applied, except for the leg (from knee to foot) and with a 10° angle of inclination on the femur. The forces involved in the knee are represented uniformly as R (Figure 2 and Figure 3). Figure 2 displays the general scheme used for kinematic calculations, and the moments of force (torque) involved in the deep squat position and the vectors in the X-Y space of the forces involved in the same position are represented in Figure 3.


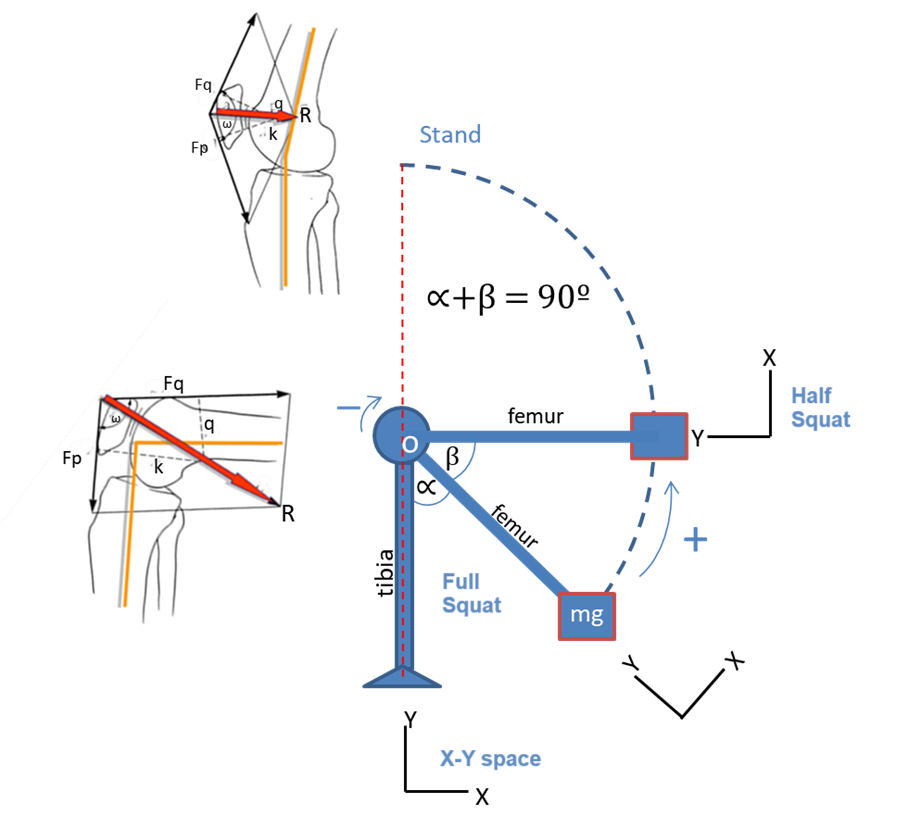


**Figure 2**. Schematic representation depicting the angular relationship between the full squat (FS) and half squat (HS) positions of the femur in a sagittal projection. This includes the virtual location of body weight (mg), the femur-tibia angle, and the X-Y spaces at the FS and FS positions. A basic outline derived from a reference subject (70 kg body weight) has been included to illustrate the vector resulting from the stress tension exerted by the patella on the femoral condylar surface during the FS exercise.


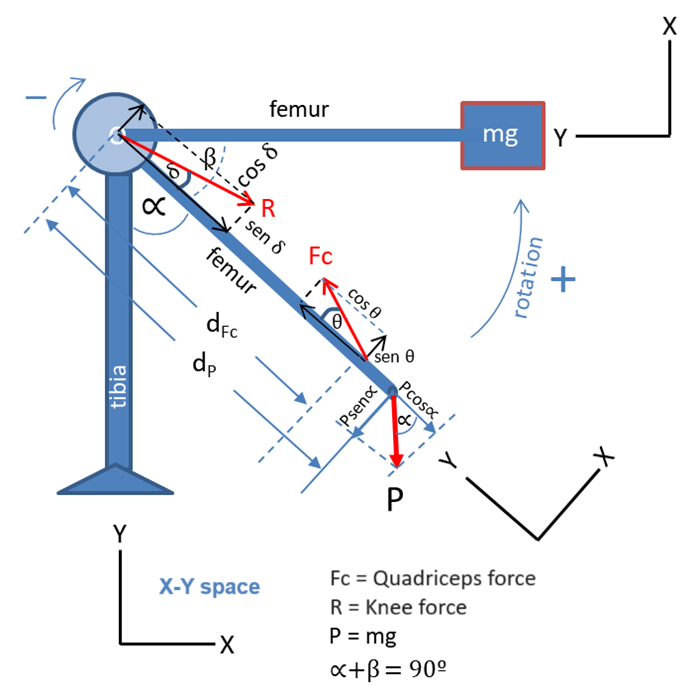


**Figure 3**. Illustration describing the forces involved in the full squat and the half squat. In the full squat, an alpha flexion angle of 40º is depicted, while in the half squat, this angle is 90º. For clarity, the body weight (P) is concentrated at the end of the femur (origin of the red arrow P). The vectors determining the value of P are Psenα and Pcosα, and those determining the force of the quadricepital muscles (Fc) are Fc·sen θ and Fc·cosθ, while those of knee force (R) are R·senδ and Rcosδ. The distance between the point of application of body weight and the center of rotation of the knee joint is represented as d_P,_ and the distance from the proximal insertion of the femur to the center of rotation of the knee joint is described as d_Fc_.

**Calculations and estimated force values**

Body weight percentages of some body structures based on Boron & Boulpaep (Boron & Boulpaep, 2017):

- HEAD = 7% Body Weight
- ARMS = 12% Body weight
- TRUNK = 46% Body weight
- LEGS = 35% Body weight
  - - Thighs (2/3 full legs) = 23% body weight
    - Legs (knee-to-foot) = 12% body weight (not used in this calculation)

The sum of the force moments applied to the femur (see Figure 3) in the deep squat position should be 0 N·m

**Moment of muscle strength** (Hr) generated by the quadriceps muscle:

Σ M0 = dFc · Fc · sen θ – d_P_· P·senα = 0, Moment of $Fc=\frac{d_{\text{P}}}{d_{Fc}}\cdot\frac{sen\alpha}{sen\theta}\cdot P$ (1)

**Force vectors on the X and Y axes**

ΣFx = 0, R sin δ + Fc·sen θ – P·sen α = 0, R sin δ = P·sen α – Fc·sen θ (2)

ΣFy = 0, -R·cos δ + Fc·cos θ – P·cos α = 0, R·cos δ = Fc·cos θ – P·cos α (3)

Dividing (2)/(3),

$$tg\delta=\frac{d_{\text{P}}}{d_{Fc}}\cdot\frac{P\cdot\cos\alpha-Fc\cdot sen\theta}{Fc\cdot\cos\theta-P\cdot sen\alpha}$$

$$R=\frac{Fc\cdot sen\theta-P\cdot\cos\alpha}{sen\delta}$$

Assigning specific values:

- Body weight 70 kg
- α = 45º
- θ = 10º
- P = m.g, m = 61.6 kg, P = 603.68 N, R = force in the suprapatellar tendon
- dFc = 0.30 m
- dP = 0.36 m

Results: Fc = 3020 N, δ = -2.23º. The negative sign indicates the direction of the vector R, in this case it rotates clockwise (initially we assign positive values for counterclockwise rotation and negative values for clockwise rotation). The angle δ indicates the direction of the vector R of the schema in **Figure 3**. Note that the arrowhead should actually be below the femur in the FS position, something that is not known until the numerical calculations are performed. Hence also the negative sign of the scalar of R. The same thing, but with a different value, happens in the half squat.

R= -2385 N

Assigning to α = 90º (half squat)

Fc = 4235 N, δ = -1.66º R = -4136 N

As indicated above, the calculations provide us with a value of Fc = 3020 N and an angle δ = -2.23° (the negative sign denotes the direction of vector R, indicating clockwise rotation in this instance. Initially, positive values were assigned for counterclockwise rotation and negative for clockwise rotation). The angle δ signifies the direction of vector R in the diagram depicted in Figure 3. It's important to note that the arrowhead should ideally be positioned below the femur in the deep squat position, a detail that becomes apparent only upon numerical calculations. Hence, the negative sign of R, which is R= -2385 N The same holds true for the half squat, although with different values: Fc = 4235 N, δ = -1.66º, R = -4136 N. It's worth noting that applying forces at a 90° angle yields higher force values and lower angles of application for R.

Based on the quantitative approximation presented above, several deductions could be made:

1. During the full squat, the largest condylar surface encounters the menisci, resulting in the load being distributed over a broader cartilaginous area.
2. The strength of the quadriceps muscles in the deep squat is measured at 3020 N, whereas in the half squat, it amounts to 4235 N.
3. The resultant force at the knee joint during the deep squat is calculated as 2385 N (the negative sign indicates direction, not scalar value), whereas in the half squat, it reaches 4136 N.

Hence, it follows that in a full squat position, there is less force available to lift the body weight compared to a half squat. The inability to lift absolute weights (kg) as high in the deep squat, in contrast to the half squat, serves as a safeguard for knee joint function. Moreover, the contact of a larger condylar surface during the deep squat facilitates better weight distribution across the knee joint. Relative body weight (BW) values range from 2 BW in the deep squat to 6 BW in the half squat (Escamilla, 2001). However, it seems that the stresses on the patella increase with greater knee flexion, potentially posing a risk to the patellar cartilage (see Figure 2). Nevertheless, when full squat exercises are performed on healthy knees with progressive loads, the adaptive response results in thickening of the patellar cartilage and an overall increase in patellar thickness (Hartmann et al., 2013). This adaptive mechanism contributes to enhanced joint function and a reduced risk of injury.

**References**

Boron, W., & Boulpaep, E. (2017). *Medical Physiology*. Elsevier Publishing.

Escamilla, R. F. (2001). Knee biomechanics of the dynamic squat exercise. *Med Sci Sports Exerc*, *33*(1), 127-141. <https://doi.org/10.1097/00005768-200101000-00020>

Hartmann, H., Wirth, K., & Klusemann, M. (2013). Analysis of the load on the knee joint and vertebral column with changes in squatting depth and weight load. *Sports Med*, *43*(10), 993-1008. <https://doi.org/10.1007/s40279-013-0073-6>
